# Invasive Fungal Infections 2021

**DOI:** 10.3390/jof8080760

**Published:** 2022-07-22

**Authors:** Immaculata Xess, Livio Pagano, Yubhisha Dabas

**Affiliations:** 1 Department of Microbiology, All India Institute of Medical Sciences, New Delhi 110029, India; yubhi.aiims@gmail.com; 2 Department of Hematology, Catholic University of Sacred Heart, 20123 Rome, Italy

Invasive fungal infections (IFIs) represent a significant problem in a large proportion of immunocompromised individuals and critically ill patients. Over the past four decades, IFIs, such as invasive aspergillosis (IA), invasive mucormycosis, invasive candidiasis and invasive cryptococcosis, have assumed greater significance, primarily because of the increased number of patients subjected to severe immunosuppression [1]. The list of opportunistic fungi causing serious, life-threatening infection increases every year and now include yeasts other than *Candida* species, hyaline molds and the pigmented or phaeoid fungi. The prevalence of IFIs in transplant and hematological malignancy patients is around 8% [2]. In ICU settings, IFIs are reported to be caused by yeasts (<2%) more often than filamentous fungi (<0.5%) [3].

Because of the costs involved, there exists great disparity in the world regarding available diagnostic tests. Microscopy and cultures correlated with clinical data can aid in forming IFI diagnosis in most situations [4]. Additionally, there are constant improvements being made in diagnostics, including the detection of several biomarkers from non-invasive samples. However, it is an area that needs to be researched further as there are many existing challenges, including sample collection from patients with debilitating conditions, subclinical presentation, microscopy and culture finding correlation, time to diagnosis and cost effectiveness. A delay or misdiagnosis can impede prompt initiation of antifungal therapy, which then can lead to poor outcomes.

There are many international guidelines for IFI diagnosis (European Organization for Research and Treatment of Cancer/Mycoses Study Group (EORTC/MSG) 2008 and 2020 [5,6]; AspICU criteria [7] for clinically suspected IA in ICUs) and treatment (Infectious Diseases Society of America (IDSA) and European Society for Clinical Microbiology and Infectious Diseases and European Confederation of Medical Mycology (ESCMID/ECMM)) [8,9,10,11]. Their role has yet to be regularly assessed for all patient groups.

Antifungal prophylaxis or empirical therapy is often practiced. However, breakthrough IFIs have been noticed in those receiving prolonged antifungal prophylaxis, such as echinocandins in hematopoietic cell transplantation [12] and posaconazole in acute myeloid leukemia (AML) patients [13]. Thus, the application of prophylactic antifungals should be reviewed with the local epidemiology data to avoid any increase in acquired resistance. Additionally, there are noted interactions between azoles and the new neoplastic drugs, which should also be taken in account [14].

For invasive candidiasis, the treatment guidelines include recommendations on antifungal duration, intravascular catheters, infective foci (deep/metastatic) investigations and the correlation of clinical and microbiological outcomes [8]. The drugs of choice are echinocandins, with few exceptions [15]. However, for *C. auris*, combinational therapies should be explored further as this, being a nosocomial pathogen, is more difficult to treat. Overall, there has been reported resistance to fluconazole, but generally, candidemia isolates are sensitive to echinocandins and amphotericin B [8,15].

For invasive aspergillosis, the current IDSA treatment guidelines detail antifungal ancillary treatments and also the duration of treatments [10]. Voriconazole is considered as the drug of choice for primary therapy in IA (especially with cases of invasive pulmonary aspergillosis), whereas L-AMB, caspofungin and posaconazole are to be preferably used as the salvage therapy drugs. Mostly, drug resistance has been reported with azoles (itraconazole, voriconazole and posaconazole), especially in *Aspergillus fumigatus*, and is said to be mostly linked with increased environmental exposure to azole fungicides [16,17,18].

For Cryptococcosis, the main clinical life-threatening presentations are cryptococcal meningoencephalitis and disseminated disease; the drugs of choice are described in detail in a different set of patients based on the host factors, and the durations of therapies are also defined [19,20]. Amphotericin B (and its lipid formulations) with flucytosine is indicated as induction therapy in HIV-infected individuals, organ transplant recipients and non-HIV, non-transplant patients, with differences in dosage and duration. The maintenance and consolidation therapy is fluconazole. However, for patients with CD4 count >100 cells/µL and undetectable viral load for >3 months, a minimum of 1 year of antifungal therapy is recommended [9]. There has been resistance reported in *C. neoformans* var *grubii* strain to fluconazole [21]. However, overall, they are susceptible to new triazoles, mainly voriconazole, posaconazole and isavuconazole [19,20,21].

Invasive mucormycosis is another dangerously disseminated infection, with the most common presentation being rhino-orbital [20]. Diagnosis is extremely important in cases of mixed infections. Antifungal treatment strategies are generally associated with surgical debridement for these cases. The focus is on the roles of amphotericin B formulations, posaconazole, combination therapies and newer therapeutic approaches with isavuconazole. Identification to the genus/species level is important since *Cuninghamella, Absidia* and *Rhizopus oryzae* may be drug-resistant both in vitro and in vivo [11,22].

To summarize, there are multiple considerations that underscore the importance of understanding both the epidemiology and resistance profile of these isolates from IFI cases. They are mostly present in immunocompromised patients where treatment modalities have to be improved on the basis of an individual patient’s conditions. It is alarming as there is a significantly very high mortality rate (40–100%) associated with IFIs. There is an increasing rate of resistance to antifungal drugs for each kind of IFI, which makes treatment success even harder to achieve. In our view, the role of antifungal agents in successful IFI treatment has to be further substantiated with the incidence of drug resistance in different patient groups.

This Special Issue focuses on the latest research in IFIs with Hanai Y et al., 2021, highlighting that voriconazole trough concentrations of ≥1.0 μg/mL significantly decrease the all-cause mortality rate in adults with IFIs and Dabas Y et al., 2022, presenting a comprehensive picture with the shift in IFI epidemiology and also raising the concern of high MICs to azoles. We thank all the authors who have contributed to the Special Issue, which we are sure will be beneficial to the readership of the journal.
